# Supplementary material for: pH-Responsive Liposome–Hydrogel Composite Accelerates Nasal Mucosa Wound Healing
Source: Pharmaceutics. 2025 May 24;17(6):690. doi: 10.3390/pharmaceutics17060690 (PMC12195687; doi:10.3390/pharmaceutics17060690)
Supplement: Supplementary file 1 [file pharmaceutics-17-00690-s001.zip › pharmaceutics-3543196-supplementary.pdf]

Supplementary materials

pH-Responsive Liposome-Hydrogel Composite Accelerate Nasal Mucosa Wound Healing

Yingchao Yang [1,2], Jingyi Chen [1,2], Shengming Wang [1,2], Yaxin Zhu [1,2], Yao Wang [1,2], Yan Chen [1,2], Mingjiang Xia [1,2], Ming Yang [3], Hongliang Yi [1,2,*] and Kaiming Su [1,2,*]

1 Department of Otorhinolaryngology Head and Neck Surgery, Shanghai Key Laboratory of Sleep Disordered Breathing, Shanghai Sixth People's Hospital Affiliated to Shanghai Jiao Tong University School of Medicine, 600 Yishan Road, Shanghai, China

2 Otolaryngology Institute of Shanghai Jiao Tong University, 600 Yishan Road, Shanghai, China

3 Department of Urology, Shanghai Sixth People's Hospital Affiliated to Shanghai Jiao Tong University School of Medicine, 600 Yishan Road, Shanghai, China

* Correspondence: Su K., sukaiming@sjtu.edu.cn; Yi H., 105178@alumni.sjtu.edu.cn

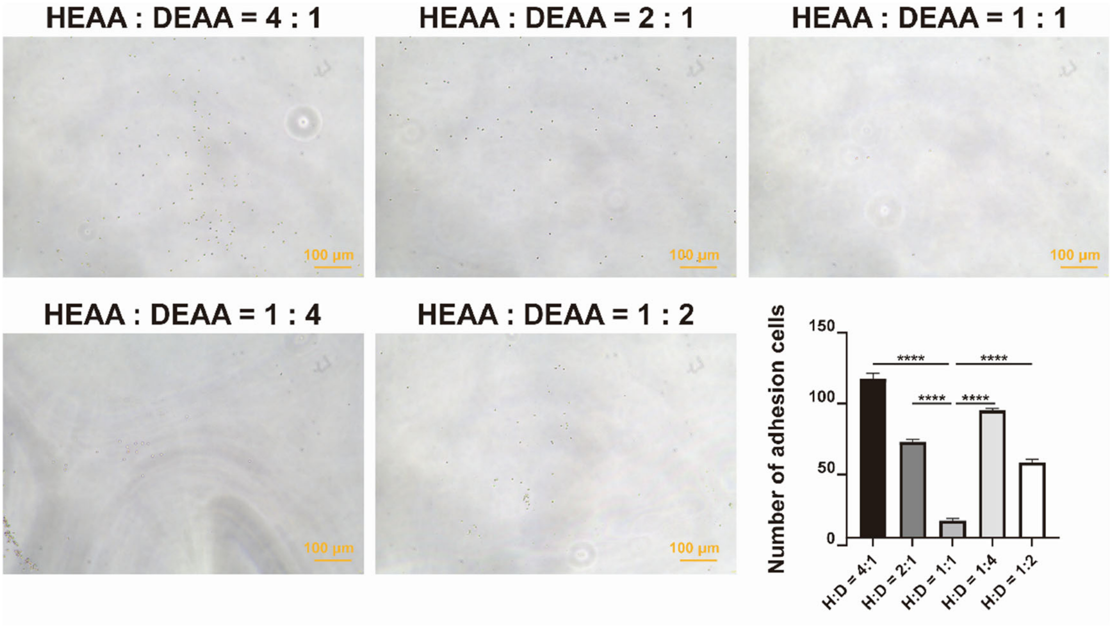

Figure S1. Cell adhesion results of hydrogels synthesized with different ratios of HEAA and DEAA (scale bar = 100 μm).

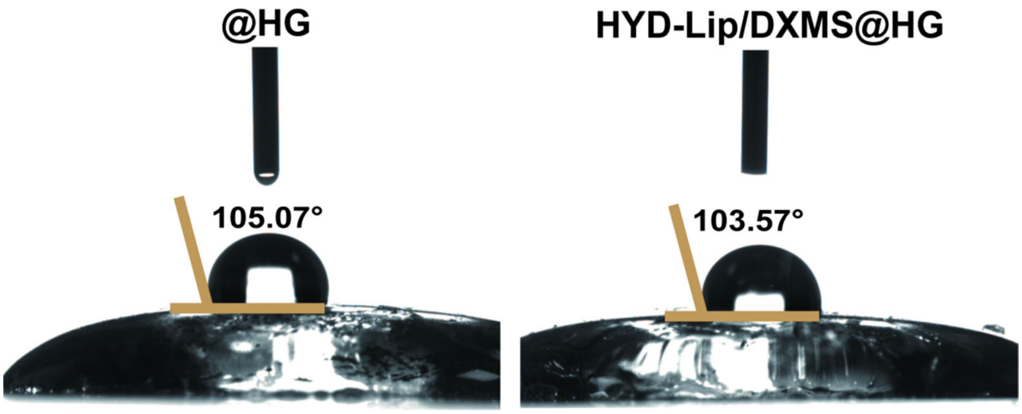

Figure S2. Water contact angle on the surfaces of @HG and HYD-Lip/DXMS@HG.

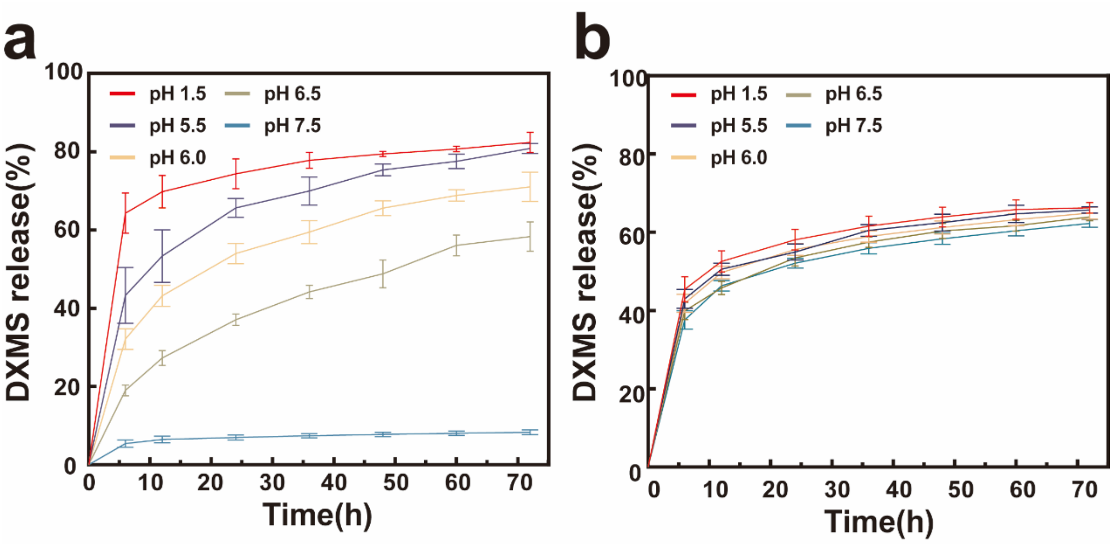

Figure S3. The cumulative release profiles of DXMS from HYD-Lip/DXMS and DXMS@HG. (a) DXMS release of HYD-Lip/DXMS in pH 1.5, pH 5.5, pH 6.0, pH 6.5, pH 7.5; (b) DXMS release of DXMS@HG in pH 1.5, pH 5.5, pH 6.0, pH 6.5, pH 7.5.

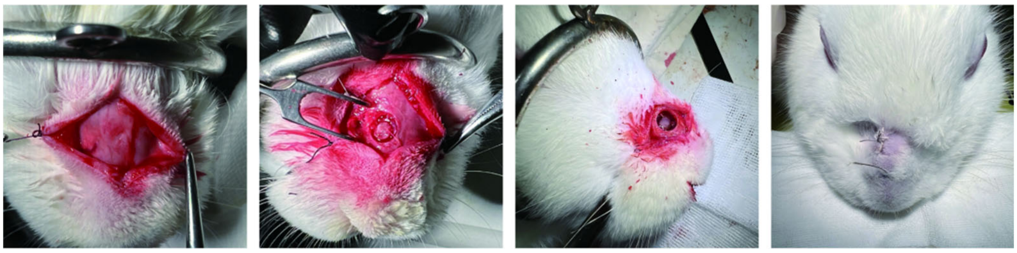

Figure S4. Schematic diagram of the nasal operation.

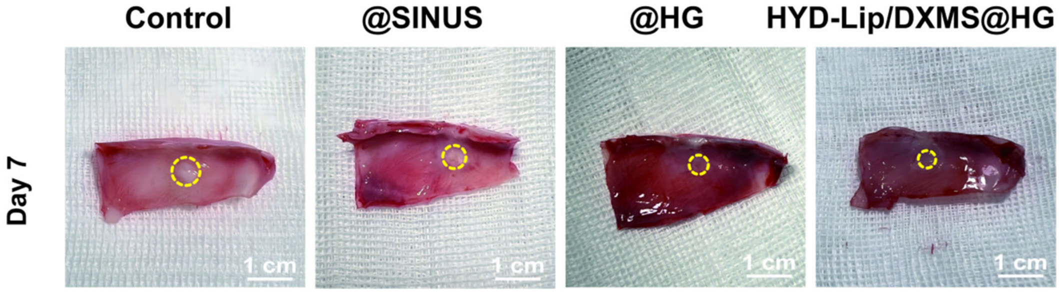

Figure S5. Repaired mucosae obtained from different treatment groups on day 14. Scale bar: 1 cm.

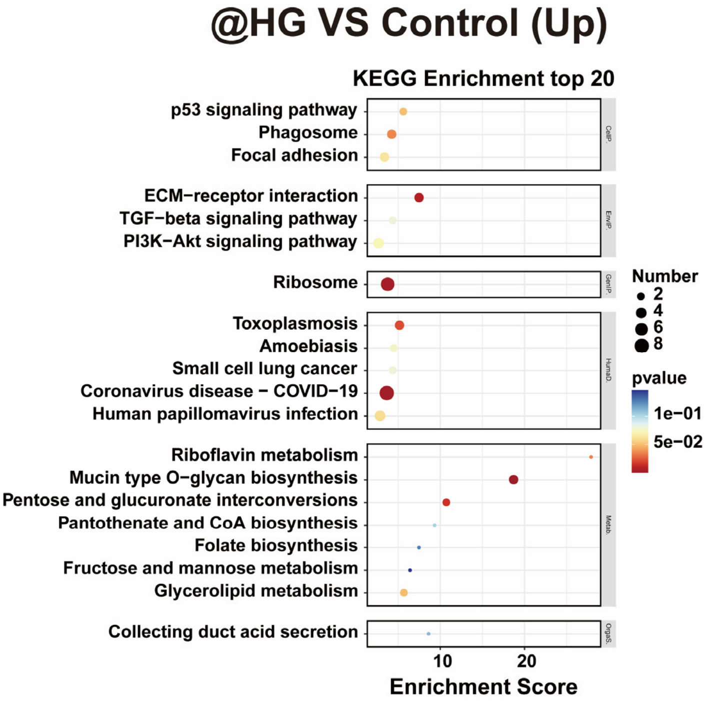

Figure S6. Top 20 upregulated/downregulated signaling pathways in the KEGG enrichment analysis between @HG and Control group.

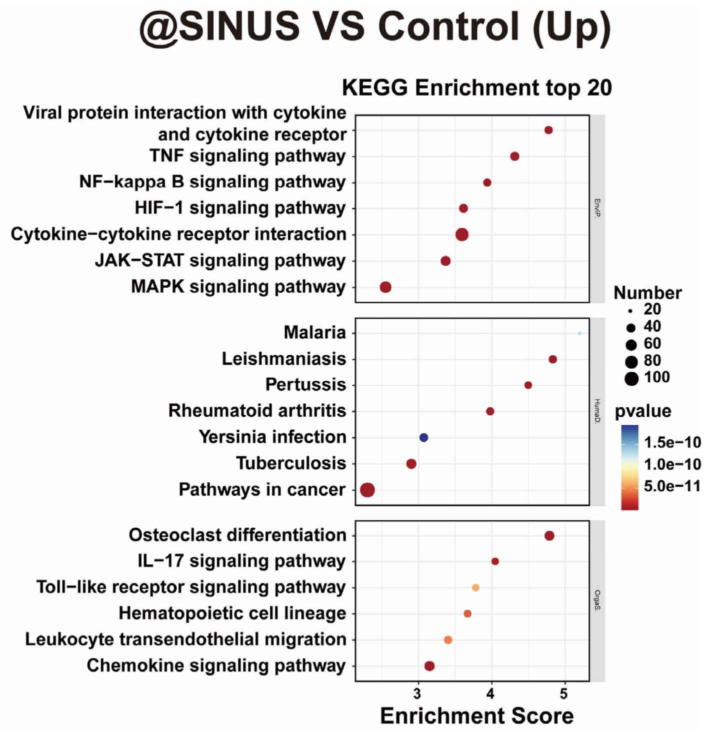

Figure S7. Top 20 upregulated/downregulated signaling pathways in the KEGG enrichment analysis between @SINUS and Control group.

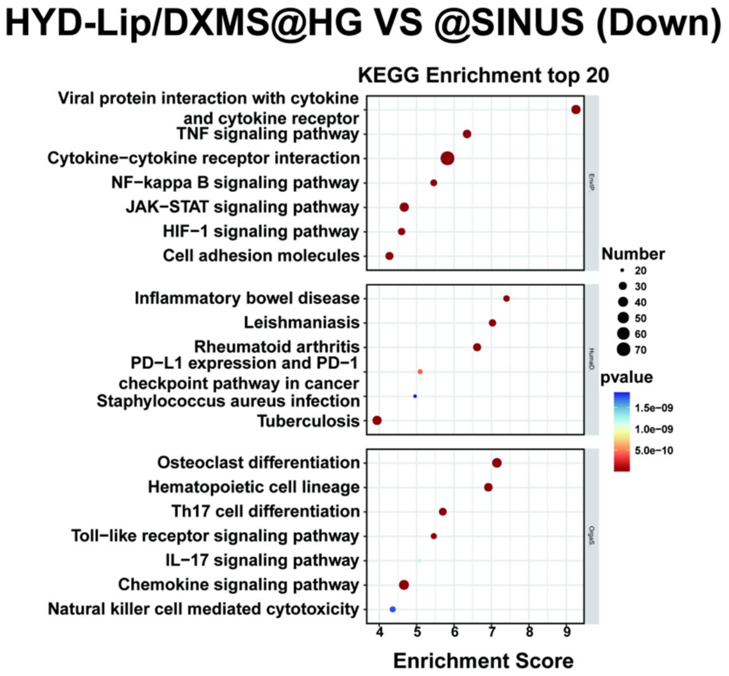

Figure S8. Top 20 upregulated/downregulated signaling pathways in the KEGG enrichment analysis between HYD-Lip/DXMS@HG and @SINUS group.

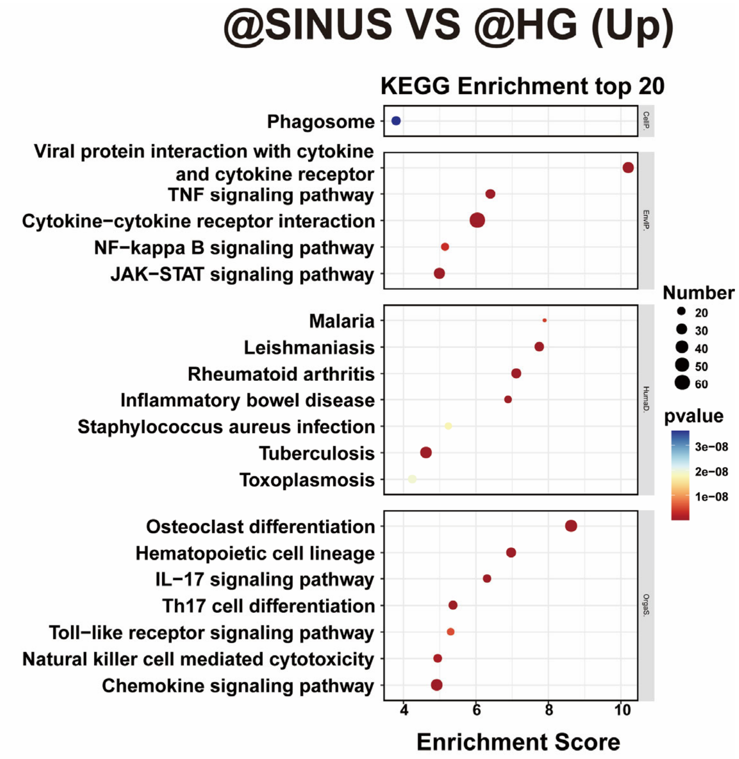

Figure S9. Top 20 upregulated/downregulated signaling pathways in the KEGG enrichment analysis between @SINUS and @HG group.
